# Can Natural Products be Used to Overcome the Limitations of Colorectal Cancer Immunotherapy?

**DOI:** 10.3389/fonc.2022.884423

**Published:** 2022-05-04

**Authors:** Jiahuan Dong, Yufan Qian, Guangtao Zhang, Lu Lu, Shengan Zhang, Guang Ji, Aiguang Zhao, Hanchen Xu

**Affiliations:** ^1^Institute of Digestive Diseases, Longhua Hospital, Shanghai University of Traditional Chinese Medicine, Shanghai, China; ^2^Department of Oncology, Longhua Hospital, Shanghai University of Traditional Chinese Medicine, Shanghai, China

**Keywords:** colorectal cancer, immunotherapy, immune checkpoint inhibitor, natural products, immunomodulation

## Abstract

Colorectal cancer (CRC) is a common cancer of the digestive system that endangers human health. Immunotherapy is widely used in the treatment of patients with cancer. Some patients with dMMR/MSI-H CRC benefit from treatments that use immune checkpoint inhibitors, but most CRC patients are not sensitive to immunotherapy. Furthermore, internal resistance and immune escape lead to a reduced immunotherapy response. Therefore, the development of an effective combination therapy to improve the response rate to immunotherapy is a goal of cancer research. Natural products are potential candidates for comprehensive cancer treatments due to their wide range of immunomodulatory effects through multifactorial underlying mechanisms. In this review, we summarize the challenges in the treatment of CRC and assess the immunomodulatory effects of natural products and their active components. Our work suggests that natural products represent potential options for combined CRC immunotherapy.

## Introduction

The incidence rate and mortality rate of colorectal cancer (CRC) are third and second among all diseases, respectively, and CRC is characterized by a lack of obvious symptoms in the early stage and poor prognosis in the advanced stage (1). The main treatments for CRC are surgery, chemotherapy, radiotherapy, and targeted therapy. The emergence of immunotherapy has provided a transformative new method for the comprehensive treatment of cancer. An important function of the human immune system is to recognize and eliminate tumor cells, a process known as tumor immune surveillance, which is mainly performed by antigen-presenting cells, T lymphocytes, B lymphocytes and natural killer (NK) cells (2, 3). Cancer cells inhibit the body’s immune system in various ways to avoid the surveillance of the immune system, resulting in tumor immune escape (4, 5). Tumor immunotherapy is a treatment method used to control and eliminate cancer cells by restarting and maintaining the tumor immune cycle and restoring the body’s normal antitumor immune response. Tumor immunotherapy mainly involves immune checkpoint inhibitors (ICIs), cellular immunotherapy and cancer vaccines. At present, the administration of ICIs is the most widely used tumor immunotherapy method. Among ICIs, the representative (PDCD1,PD-1) inhibitor, its (CD274,PD-L1) inhibitor, and cytotoxic T-lymphocyte associated protein 4 (CTLA4) restore the ability of immune cells to fight tumors by counteracting the inhibition of the immune system by tumor cells. At present, several ICIs targeting PDCD1(PD-1), CD274(PD-L1) and CTLA4 have been approved for the clinical treatment of various solid tumors, including MSI-H/dMMR CRC (4, 6, 7). However, there are still many challenges in the treatment of CRC with ICIs. MSI-H/dMMR tumors account for 5% of CRC cases, and some patients can benefit from ICIs, but most CRC tumors are still in a “cold” state. Therefore, it is necessary to find methods to transform “cold” tumors into “hot” tumors to make them more sensitive to ICIs.

Natural products, including plants, mushrooms, bacteria, animal metabolism products or organs and even mineral substances, characterized by various structure and activities, are well-known by the researchers gradually in recent years. Some evidence show that natural products have potential immunomodulatory effects and can play a synergistic role when combined with ICIs. So it is more important to explore the mechanisms of nature products for providing strongly clinical evidence. In this review, we summarized the effects of natural products on modulating macrophages, T cells, NK cells and a combination of ICIs.

## The Advantages and Limitations of ICIs for CRC Treatment

Currently, the main treatment for resectable CRC is surgery combined with chemotherapy or targeted medicines. However, metastatic CRC treatments remain challenging. According to retrospective analyses, some patients benefit less from 5-FU adjuvant chemotherapy (8, 9) because the molecular mechanism of CRC is different, and it may lead to more heterogeneity.

In 1997, the National Cancer Institute first defined microsatellite instability (MSI), which is a form of genomic instability associated with defective DNA mismatch repair (dMMR) in tumors; two mononucleotide repeats (BAT26 and BAT25) and three dinucleotide repeats (D5S346, D2S123, D17S250) were validated in the detection panel (10). MSI can currently be assessed by immunohistochemistry (IHC), including the expression of MSH2, MSH6, PMS2, MLH1 and polymerase chain reaction (PCR); moreover, novel next-generation sequencing (NGS) has become a new testing option. Intriguingly, investigators found that MSI-high (MSI-H) CRC is associated with increased neoantigen load and immune infiltration (11–14), which means that immune checkpoint blockade may be an effective method of therapy.

Currently, MSI assessment can influence the selection of clinical medications and predict outcomes in colorectal cancer (15, 16). KEYNOTE-164, a phase II clinical trial, demonstrated that pembrolizumab was effective in MSI-H-dMMR CRC; it displayed a higher overall response rate (ORR) and improved progression-free survival (PFS) (17). KEYNOTE-177, which enrolled patients with stage IV MSI-H-dMMR CRC, demonstrated that the PFS in the pembrolizumab group was prolonged by 8.3 months, with an ORR of 43.8%, and there were fewer treatment-related adverse events in the pembrolizumab group than in the chemotherapy group (18, 19). The PDCD1(PD-1) inhibitor nivolumab showed durable responses and disease control, and 51 patients with metastatic MSI-H CRC had disease control for 12 weeks or longer; these results were similar to the findings of the CheckMate-142 study (20). Meanwhile, nivolumab plus the CTLA4 inhibitor ipilimumab displayed effective responses: 80% of the 119 patients had a disease control rate over 12%, and the investigator-assessed ORR was 55% (21). Considering the dose-dependent effect of ipilimumab, nivolumab combined with a low dose of ipilimumab showed robust and durable clinical benefit, with a 69% ORR and 84% disease control rate until the data cutoff (22). Based on the results of numerous clinical trials, the 2021 NCCN guidelines suggest that patients with advanced or metastatic CRC can use immunotherapy checkpoint blockade for subsequent therapy.

Although the results of clinical trials on dMMR/MSI-H are exciting, the response rates range between 30% and 50%, suggesting that resistance and immune escape still exist (23–25). More clinical trials have focused on the combination of VEGF inhibitors and chemotherapy (26–28); most of the studies are ongoing and the results are pending.

## How to Make ICIs More Effective for CRC

The incidence of dMMR CRC is approximately 5%, which is far lower than that of proficient mismatch repair (pMMR) CRC (29). The conventional treatment of pMMR/microsatellite stable (MSS) CRC is systematic chemotherapy based on 5-fluorouracil and platinum, and most of patients have lower responses to immunotherapy due to intrinsic resistance. The mechanism may involve low TMB, a lack of tumor antigens and a suppressive microenvironment (30, 31). How to turn “cold” tumors into “hot” tumors is currently a popular topic in academic research.

Some studies have shown that chemotherapy can improve immunogenicity and enhance the efficacy of ICIs (32, 33). Currently, some ongoing clinical trials are exploring chemotherapy combined with angiogenesis medicine and ICIs. A protocol for unresectable metastatic CRC was described in the AtezoTRIBE study that enrolled patients receiving FOLFOXIRI plus bevacizumab; some patients received sequential atezolizumab (34). A study of chemorefractory MSS mCRC included two cohorts: one was pembrolizumab plus pemetrexed, and in the other oxaliplatin was added for the dose escalation portion of the study (35). Although the trials have not shown the endpoint and some did not consider the microsatellite status, they represent worthwhile attempts.

Meanwhile, antiangiogenic and multitarget drugs also display synergistic sensitivity to ICIs. Innate immunity and immune adoption can directly lead to tumor angiogenesis (36), and the most widely studied VEGF family also drives angiogenesis to promote immune escape and immune suppression (37). Bevacizumab is the first antiangiogenic drug approved for metastatic CRC, NSCLC, metastatic renal cell carcinoma, and recurrent/metastatic cervical cancer (38, 39). Combining bevacizumab with immunotherapy promoted T cell infiltration, enhanced local immune activation and inhibited the expansion of MDSCs in preclinical studies (40, 41). Likewise, the use of bevacizumab combined with ICIs has been studied in many clinical trials, such as those investigating NSCLC, recurrent glioblastoma (42) and ovarian cancer (6, 43, 44); clinical trials for CRC are still ongoing, especially for MSS/pMMR CRC (45–47). In addition, multitarget antiangiogenic drugs show better responses. An open-label, phase II trial that enrolled 25 CRC patients demonstrated that a dose of regorafenib 80 mg can increase sensitivity to nivolumab, and the median PFS was 7.9 months (48). Similarly, a case report showed that in an MSS patient who received fruquintinib plus sindilizumab for six cycles, the lymph nodes became fewer and smaller, and CA199 was decreased (49). In a phase II study of patients with advanced refractory CRC, the median OS was 6.6 months for patients who received durvalumab and tremelimumab compared with the cohort who received supportive care, and the patients accepted the continuation of treatment with TAS-102 or regorafenib after disease progression (50). Not only the clinical report but also the author-selected syngeneic MSS model demonstrated that the combination of the two drugs could inhibit proliferation, induce apoptosis and promote vascular normalization (49). Hence, ICIs combined with antiangiogenic drugs may be a promising method of MSS CRC treatment.

In addition, CRC has some mutations driven by RAS, BRAF, EGFR, HER-2 and POLE, and most of them can impact treatment and prognosis. For example, approximately 10% of patients diagnosed with CRC harbor the BRAF mutation, which is considered a poor prognostic factor, and one third of mutations are associated with MSI (51–54). As shown in the Checkmate-142 study, the ORR was 25% in the BRAF mutation group and similar to that in the combined nivolumab and ipilimumab group (21, 22). A case report suggested that a patient harboring MSS and BRAF V600E mutations responded well to nivolumab and bevacizumab, achieving more than 17 months of PFS (55). However, some studies have reported that the BRAF mutation does not influence the response to immunotherapy (56, 57), and combination therapy needs to be further explored in large samples. Apart from the BRAF V600E mutation, a common oncogenic mutation is RAS mutation, especially KRAS mutation, which accounts for approximately 40% of CRC cases, and is related to poor prognosis and metastasis (58, 59). In addition, the KRAS mutation in CRC is associated with immune suppression and immune infiltration (60, 61). Some current clinical trials are aimed at investigating these mutations. A phase I/II study enrolled CRC patients with RAS mutations regardless of MSS status to assess the safety and efficacy of the combination of durvalumab and tremelimumab (62). Likewise, a phase II study focused CRC with RAS or BRAF mutations and investigated the use of nivolumab combined with FOLFOXIRI/bevacizumab (63). These studies demonstrate that immunotherapy still has considerable potential for the treatment of CRC mutations.

## Natural Products Exert Modulating Effects on the Immune System in CRC

In addition to using the above-mentioned methods, researchers have paid attention to natural products. Natural products include the active compounds in plants, mushrooms, bacteria, animal metabolism products or organs and even mineral substances. These products have been explored and used for a thousand years. Some active compounds have proven antitumor, antioxidant, and anti-inflammatory effects (64). However, there are still a number of natural products that have adverse or toxic effects; these products must be used properly or avoided. The specific mechanism of natural products is still unknown and requires further investigation. According to recent evidence, natural products can directly regulate innate immunity and adoptive immunity (65); they play a role in preventing tumor development and modulating immunity (66–68). Thus, natural products show promise as agents in immunotherapy.

First, natural products can influence the immune microenvironment of early CRC in multiple ways, affecting M2 macrophage polarization to M1 to exert an immunomodulation effect. Isoliquiritigenin, a flavonoid derived from licorice, blocks M2 polarization in colitis-related tumorigenesis and inhibits the development of colorectal cancer by downregulating PGE2/IL6 signaling (69). Apple polysaccharides not only prevent the carcinogenesis induced by AOM/DSS in mice but also modulate the M2 to M1 macrophage phenotype and upregulate TLR4/NF-κB signaling (70). Most basic experiments have adopted the AOM/DSS model or the CAC model to indicate the mechanism by which natural products affect macrophages (70–75). Taken together, these results show that there are many natural products that play important roles in inflammatory cancer transformation *via* different mechanisms, and natural products will intervene in CRC development in the near future.

Natural products can also influence T cells, NK cells and Treg cells. Black raspberries can significantly inhibit CRC progression and increase NK cells in tissues infiltrating the APC ^Min+/-^ DSS and AOM/DSS models, and the results were validated in human CRC tissue (76, 77). In addition, Ecklonia cava fucoidan (ECF) not only stimulates NK cell activation and proliferation but also induces NK cell activation through DCs (78). Moreover, rice hull polysaccharides (RHPS) can enhance NK cell activation and induce the secretion of INFG (INF-gamma) and TNF(TNF-alpha) *in vitro*; they also inhibit tumors in CT-26-bearing mice and enhance NK cell activation *in vivo (* 79). It is clear that natural products demonstrate antitumor effects by influencing NK cell activity.

Similarly, natural products can cause T cells to exert immune modulating effects. Another well-known immunomodulatory natural product, curcumin, may suppress the expression of FOXP3 on Tregs and enhance the ability of T cells to kill tumor cells and modulate multiple immune cytokines (80–83). A control study revealed that the administration of curcumin can suppress the transcription of the FOXP3 gene and convert Tregs to Th1 cells by enhancing INFG (INF-gamma) production (84). In an *in vivo* experiment, researchers selected a CT-26 mouse model to compare curcumin and sildenafil combined with anti-PDCD-1(PD-1) and showed that the tumor volume was smaller in the combined treatment group (85). Based on preclinical research, curcumin is a potential natural product, especially when combined with immunotherapy. In addition, natural products can inhibit CRC metastasis by regulating the tumor microenvironment. The natural small molecule bigelovin may inhibit colorectal tumor growth by regulating the tumor immune microenvironment, increasing the T lymphocyte and macrophage populations, and inhibiting liver and lung metastasis of CRC through the IL6/STAT3 pathway (86). Furthermore, natural products can also upregulate IL-17 secretion to stimulate T cell proliferation or differentiation. Gan cao (Glycyrrhiza uralensis Fisch.) polysaccharides, especially those of low molecular weight, can upregulate IL-17 and enhance T lymphocyte proliferation (87). The author found that red wine extract could inhibit tumor progression and affect T lymphocyte cell differentiation into T helper 17 cells (88). Other studies have shown that natural products can alleviate tumor growth and modulate immunity by restoring intestinal barriers (89), inducing DC maturation (90) and reducing the accumulation of myeloid-derived suppressor cells (MDSCs) (91). It’s noteworthy that quercetin and alantolactone not only can induce immunogenic cell death and cell apoptosis for MSS CRC, but also can reduce immunosuppressive cell population like MDSCs, Treg and so on. This study adjusted nanoformulated codelivery, on the other hand provided a basis for multi-drug combination of nature products (92).

Natural products combined with ICIs demonstrate better responses in patients, and this strategy may be a prospective method for use in the clinic. Many studies have investigated natural products other than curcumin. Atractylenolide I significantly improves the cytotoxic effect of T lymphocytes on tumor cells and promotes the antigen presentation of tumor cells. Atractylenolide I has a synergistic effect in the treatment of CRC when combined with immune checkpoint inhibitors (93). Astragaloside IV can significantly induce M2 macrophages to M1 polarization, decrease the production of anti-inflammatory factors and increase proinflammatory INFG (INF-gamma) in colorectal tumors (94). Meanwhile, astragaloside IV combined with a PDCD1(PD-1) inhibitor exhibited a synergistic effect on inhibiting tumor growth and T cell infiltration. Inulin, which is derived from dietary fiber, can significantly improve the systemic antitumor efficacy of anti-PDCD-1(PD-1) therapy and effectively slow tumor growth by altering the gut microbiome. Compared with anti-PDCD-1(PD-1) alone, the synergistic use of inulin and anti-PDCD-1(PD-1) significantly increased CT-26 GP70-specific CD8+ T cells in mice. Interestingly, by transforming inulin into inulin gel before its use in combination with anti-PDCD-1(PD-1), the effect was improved (95). This suggests that natural products have potential regarding changes in the forms of administered medicines. Many experiments have simulated the combination of natural products and ICIs (96–98). A preclinical study was conducted that explored anti-PDCD-1(PD-1) alone and in combination with natural products and anti-CTLA4. High-dose vitamin C can decrease tumor volumes combined with anti-PDCD-1(PD-1) and anti-CTLA4 and enhance CD8^+^ T cell cytotoxic activity. This research was conducted not only in CRC but also in breast cancer, pancreatic cancer and melanoma with mismatch repair-deficient tumors with a high mutational burden (99) **(** **Table 1****).**

**Table 1:** Natural products exert modulating effects on the immune system in CRC.

Nature product	Model	Results	Reference
Isoliquiritigenin	AOM/DSS mice	PGE2 ↓ IL6 ↓	(69)
	Raw267.4 cell	M2 polarization ↓	
	Mouse peritoneal macrophages	p-STAT3 ↓ iNOS ↑	
		CLCX10 ↑	
Apple polysaccharide	AOM/DSS mice	TLR-4 Myd88 p65 ↑	(70)
	Raw267.4 cell		
Vitexin	AOM/DSS mice	M1 ↑	(71)
	CAC model	TNF(TNF-alpha) IL-1β IL6 ↓	
		NO in tumor tissue ↑	
β-Carotene	AOM/DSS mice	IL6 pSTAT3 ↓	(72)
	U937 cells		
Berberine	AOM/DSS	EGFR-ERK signaling ↓	(73)
	APC ^Min/+^ mice		
	Raw267.4 cell		
Cardamonin	Raw267.4 cell	iNOS TNF(TNF-alpha)IL6 ↓	(75)
	HCT-116	NF-κB ↓	
Black Raspberries	APC ^Min/+^/DSS	NK cells infiltration ↑	(76)
	AOM/DSS		
	Human CRC tissues		
Elkonia cava fucoidan	CT-26	NK cells activation ↑	(78)
	NK cells and DCs from C57BL/6 spleen	INFG (INF-gamma) ↑	
Rice hull polysaccharides	NK-92 MI	NK cells activation ↑	(79)
	CT-26	INFG (INF-gamma) ↑	
Curcumin	Advanced CRC patients PBMC	FOXP3+Treg ↓	(84)
		INFG (INF-gamma) ↑	
Curcumin	CT-26	Tumor volume ↓	(85)
Bigelovin	CT-26	T lymphocyte	(86)
	HCT-116	Macrophage population ↑	
Glycyrrhiza uralensis Fisch.	CT-26	IL-17 ↑	(87)
		T lymphocyte proliferation ↑	
Red wine extract	CT-26	Th17 differentiation ↓	(88)
	HCT-116		
	SW620		
	MC38		
Dendrobium officinale polysaccharides	AOM/DSS	—	(89)
Maitake Z-fraction	Colon-26 mice	—	(90)
Juglone	CT-26	INFG (INF-gamma) ↑	(91)
	MDSCs	MDSCs accumulation ↓	
Quercetin and Alantolactone	CT-26	MDSCs Treg IL-10 IL-1β TGF-β CCL-2 ↓	(92)
	Orthotopic colorectal cancer model		
Atractylenolide I	MC38	CD8^+^ T cell ↑	(93)
	MC38-OVA		
	OT-1 mice		
Astragaloside IV	CT-26 mice	TGF-β IL-10 VEGF-α ↓	(94)
		INFG (INF-gamma) IL-12 TNF(TNF-alpha) ↑	
Inulin	CT-26 mice	SCFAs (feces) ↑	(95)
		Induce stem-like Tcf^+^PD-1^+^T cell	
Sanguisorbae Radix	Humanized PD-L1 MC38 mice	Infiltrated cytotoxic T cells ↑	(96)
	Recombinant Jurkat T cells		
Pectin	MC38 mice	Infiltrated cytotoxic T cells ↑	(97)
	CRC patients fecal microbiota transplantation model	Butyrate (feces) ↑	
Andrographolide	CT-26 mice	CD8^+^ and CD4^+^ T cell function ↑	(98)
		Fasl perforin ↑Granzyme B ↑	
High-dose vitamin C	CT-26	CD8^+^ T cell cytotoxic ↑	(99)
	MC38		

Natural products combined with ICIs had better results in melanoma, lung cancer and breast cancer studies (100, 101), and they can be gradually extended to the study of pancancer in the future.

## Conclusion and Perspectives

Epidemiologic evidence show that the CRC incidence is strongly related to interaction between the environment exposures and gene alternations (102). Colorectal carcinogenesis includes three major global genetic and epigenetic aberrations: chromosomal instability (CIN), CpG island methylator phenotype (CIMP) and MSI. Although gene factors may lead to the individual risk and increase hereditary susceptibility, CRC are largely affected by diet factors and lifestyle alterations (103), like smoking, alcohol, obesity and so on (104–106). A study demonstrated that high-fat-diet-induced obesity may impair CD8^+^T cell function in the murine tumor microenvironment through the metabolic pathway (107). Conversely lifestyle and diet factors can also affect gene alternation to contribute to the onset of CRC. Similarly smoking was associated with a 59% increased risk of CRC and strongly related to MSI-H and KRAS wild type CRC in a large case-control study which enrolled 4919 participants (108).

**Figure 1:**
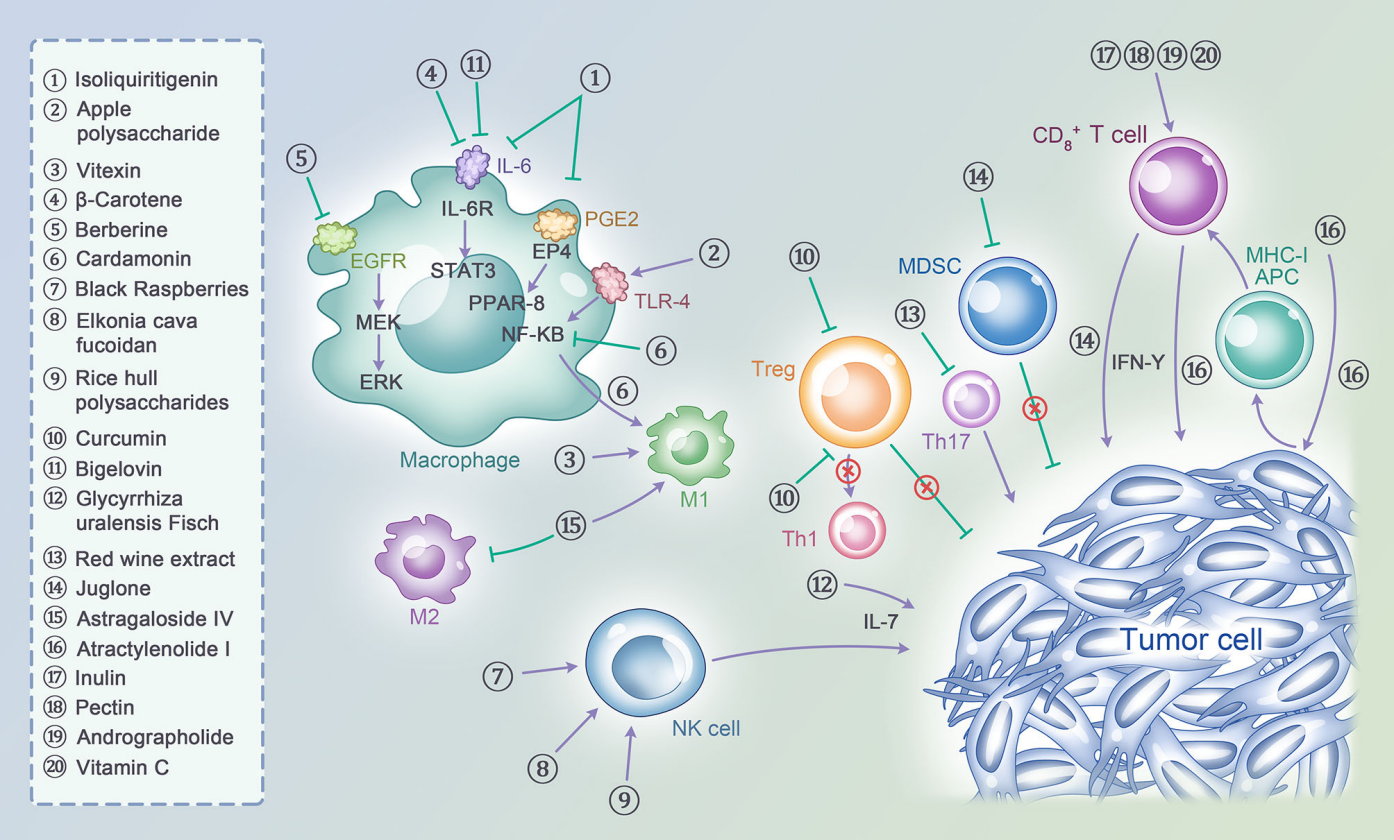
A variety of natural products act on act on multiple cell subtypes in the tumor immune microenvironment, including T cells, NK cells, Macrophages, MDSCs, Tregs and tumor cells themselves, to exert immunomodulatory effects and thus enhance the ability of anti-tumor immunity.

Base on the relationship between diet factors or lifestyle and immunity, some researchers addressed a concept of molecular pathological epidemiology (MPE) which could provide the better understanding of environment-tumor-immune interactions (109). Among them, the researches proposed several classes of substance with immunomodulatory effects in CRC, including aspirin, vitamin D, inflammatory diets and omega-3 polyunsaturated fatty acids. Take an example for omega-3 polyunsaturated, a higher intake of marine omega-3 polyunsaturated was associated with the risk of CRC with different density FOXP3+ cells (110). Since the introduction of immunotherapies, some patients have benefited, but there are still crucial problems to be solved. Preclinical studies have shown that natural products can exert antitumor effects and modulate immunity by affecting T cells, NK cells, and Tregs in CRC (**Figure 1**). If researches adopt the MPE model and integrate the immunotherapies to the model in the future, that will be a promising method which can provide more accurate strategies for the treatment, especially the field of nature products which link to the environment and immune. Natural products have some limitations; the ranges of safe doses remain undetermined and adverse effects such as hepatotoxicity and renal toxicity must be controlled. Natural products have the advantages of being easy to obtain and widely used, and they have multiple targets. Natural products have been proven effective in the early stage of CRC, especially on the transformation of adenoma to adenocarcinoma, and in advanced cancer stages, natural products can inhibit tumor progression. Meanwhile, combining natural products with ICIs can maximize the antitumor effects by acting on multiple targets.

In summary, natural products can regulate the immune system and enhance immuno-oncological effects, especially when combined with ICIs, which will be a promising strategy in the future that is gradually accepted into clinical practice.

## Author Contributions

HX and AZ proposed the topic and made the frame. JD and YQ contributed to original draft preparation. GZ, LL, and SZ participated in part of text arrangement and literature collection. LL and JD participated in the conception and drawing of the image. GJ, AZ, and HX revised the manuscript. All authors contributed to the article and approved the submitted version.

## Funding

This work was supported by National Nature Science Foundation of China, No. 81874206, 82104466; Shanghai Frontiers Science Center of Disease and Syndrome Biology of Inflammatory Cancer Transformation (2021KJ03-12); Shanghai Rising-Star Program, No. 20QA1409300; and the Program for Young Eastern Scholar at Shanghai Institutions of Higher Learning, No. QD2019034.

## Conflict of Interest

The authors declare that the research was conducted in the absence of any commercial or financial relationships that could be construed as a potential conflict of interest.

## Publisher’s Note

All claims expressed in this article are solely those of the authors and do not necessarily represent those of their affiliated organizations, or those of the publisher, the editors and the reviewers. Any product that may be evaluated in this article, or claim that may be made by its manufacturer, is not guaranteed or endorsed by the publisher.

## Abbreviations

CRC, Colorectal cancer; dMMR, defective DNA mismatch repair; MSI, Microsatellite instability; MSI-H, Microsatellite instability High; NK cell, Nature killer cell; ICIs, Immune checkpoint inhibitors; PD-1, Programmed cell death 1; CD274(PD-L1), Programmed cell death ligand 1; CTLA4, Cytotoxic T-lymphocyte associated protein 4; IHC, Immunohistochemistry; PCR, Polymerase chain reaction; NGS, Novel next-generation sequencing; ORR, Overall response rate; PFS, Progression-free survival; NCCN, National Comprehensive Cancer Network; VEGF, Vascular endothelial growth factor; pMMR, proficient mismatch repair; MSS, Microsatellite stable; TMB, Tumor mutation burden; NSCLC, Non-small-cell lung cancers; MDSC, Myeloid-derived suppressor cells; OS, Overall survival; PGE2, Prostaglandin E2; IL6, Interleukin 6; AOM, Azoxymethane; DSS, Dextran sodium sulfate; TLR4, Toll-like receptor 4; NF-κB, Nuclear factor-kappa B; INFG (INF-gamma), Interferon-gamma; TNF(TNF-alpha), Tumor necrosis factor-alpha; STAT3, Signal transducer and activator of transcription 3.
